# Complete Genome Sequence of Serratia marcescens Myophage MyoSmar

**DOI:** 10.1128/MRA.01046-19

**Published:** 2019-09-19

**Authors:** Savannah Cooper, Quang Nguyen, Heather Newkirk, Mei Liu, Jesse Cahill, Jolene Ramsey

**Affiliations:** a Center for Phage Technology, Texas A&M University, College Station, Texas, USA; Queens College

## Abstract

Serratia marcescens is an opportunistic pathogen that causes respiratory, urinary, and digestive tract infections in humans. Here, we describe the annotation of Serratia marcescens myophage MyoSmar. The 68,745-bp genome encodes 105 predicted proteins and is most similar to the genomes of Pseudomonas PB1-like viruses.

## ANNOUNCEMENT

Serratia marcescens is an opportunistic bacterium that causes urinary, respiratory, and digestive tract infections of humans (1). Many S. marcescens strains have acquired resistance to critical antibiotics through an extended-spectrum beta-lactamase (2). Bacteriophage represent a potential alternative therapeutic, and we describe here the genome of the S. marcescens myophage MyoSmar.

Bacteriophage MyoSmar was isolated from filtered water (pore size, 0.2 μm) collected at a College Station Research Park pond (College Station, TX) by its ability to form plaques on lawns of S. marcescens D1 cells (catalog number 8887172; Ward’s Science). Both the host and phage were cultured at 30°C in LB broth and agar (BD) with the soft agar overlay method described by Adams (3). Crude samples were negatively stained with 2% (wt/vol) uranyl acetate and then viewed by transmission electron microscopy at the Texas A&M Microscopy and Imaging Center to determine phage morphology (4). Genomic DNA was purified using the shotgun library preparation modification of the Wizard DNA clean-up system (Promega), and a paired-end 250-bp library was prepared using an Illumina TruSeq Nano low-throughput kit (5). Sequencing occurred on the Illumina MiSeq platform with v2 500-cycle chemistry. The 417,609 total sequence reads in the index were quality controlled with FastQC (http://www.bioinformatics.babraham.ac.uk/projects/fastqc/). Trimming occurred using the FASTX-Toolkit v0.0.14 (http://hannonlab.cshl.edu/fastx_toolkit/). Finally, a single contig with 632-fold coverage was assembled using SPAdes v3.5.0 (6). PCR across the contig ends (forward primer, 5′-ATCGCTAACTCTATCGCTTCTATC-3′; reverse primer, 5′-CCTATTCGCCCGACTCAATAAA-3′) and Sanger sequencing of the resulting product were used to confirm that the contig sequence was complete and correct. Structural annotations were completed using ARAGORN v2.36, Glimmer v3.0, and MetaGeneAnnotator v1.0 (7–9). TransTermHP v2.09 predicted Rho-independent terminators (10). Gene function predictions were made using primarily InterProScan v5.22-61 and BLAST v2.2.31, but also TMHMM v2.0 (11–13). BLAST was performed against the NCBI nonredundant database and UniProtKB Swiss-Prot and TrEMBL databases, s with a 0.001 minimum expectation value cutoff (14). All of the tools listed were used with default parameters and are in the Center for Phage Technology Galaxy and Web Apollo instances (https://cpt.tamu.edu/galaxy-pub/) (15, 16).

MyoSmar is a myophage with a 68,745-bp genome. With its 105 predicted protein-coding regions, of which only 19 were ascribed a putative function, and no tRNAs detected, MyoSmar has 92.8% coding density and a G+C content of 49%. PhageTerm predicted 3,559-bp terminal repeats, and the genome was reopened with the repeat at the left boundary (17). Based on a genome-wide comparison using progressiveMauve v.2.4.0, MyoSmar is most closely related to *Escherichia* phage ECML-117 (GenBank accession number JX128258), with 17.41% nucleotide identity and 47 similar proteins (18). Despite lower nucleotide identity, MyoSmar also shares at least 49 proteins with various Pseudomonas phages, including *Pseudomonas* phage PB1 (GenBank accession number EU716414). While the PB1-like viruses primarily infect *Pseudomonas* species, the genome organization and size similar to those of the PB1-like viruses indicate that S. marcescens phage MyoSmar may fit into this group of phages, which are reported to be isolated from and present within metagenomic data sets from diverse global environments (19).

### Data availability.

The genome sequence and associated data for phage MyoSmar were deposited under GenBank accession number MN062189, BioProject accession number PRJNA222858, SRA accession number SRR8869239, and BioSample accession number SAMN11360398.
